# Structure and computational analysis of a novel protein with metallopeptidase-like and circularly permuted winged-helix-turn-helix domains reveals a possible role in modified polysaccharide biosynthesis

**DOI:** 10.1186/1471-2105-15-75

**Published:** 2014-03-19

**Authors:** Debanu Das, Alexey G Murzin, Neil D Rawlings, Robert D Finn, Penelope Coggill, Alex Bateman, Adam Godzik, L Aravind

**Affiliations:** 1 Joint Center for Structural Genomics, La Jolla, CA, USA; 2 Stanford Synchrotron Radiation Lightsource, SLAC National Accelerator Laboratory, Menlo Park, CA, USA; 3 MRC Laboratory of Molecular Biology, Cambridge Biomedical Campus, Francis Crick Avenue, Cambridge CB2 0QH, UK; 4 Wellcome Trust Sanger Institute, Wellcome Trust Genome Campus, Hinxton, Cambridgeshire CB10 1SA, UK; 5 European Molecular Biology Laboratory, European Bioinformatics Institute, Wellcome Trust Genome Campus, Hinxton, Cambridgeshire CB10 1SD, UK; 6 Howard Hughes Medical Institute, Janelia Farm Research Campus, 19700 Helix Drive, Ashburn, VA, USA; 7 Program on Bioinformatics and Systems Biology, Sanford-Burnham Medical Research Institute, La Jolla, CA, USA; 8 National Center for Biotechnology Information, National Library of Medicine, Building 38A, Bethesda, MD, USA

**Keywords:** CA_C2195, Peptidase, DUF4910, DUF2172, HTH_47, Structural genomics

## Abstract

**Background:**

CA_C2195 from *Clostridium acetobutylicum* is a protein of unknown function. Sequence analysis predicted that part of the protein contained a metallopeptidase-related domain. There are over 200 homologs of similar size in large sequence databases such as UniProt, with pairwise sequence identities in the range of ~40-60%. CA_C2195 was chosen for crystal structure determination for structure-based function annotation of novel protein sequence space.

**Results:**

The structure confirmed that CA_C2195 contained an N-terminal metallopeptidase-like domain. The structure revealed two extra domains: an α+β domain inserted in the metallopeptidase-like domain and a C-terminal circularly permuted winged-helix-turn-helix domain.

**Conclusions:**

Based on our sequence and structural analyses using the crystal structure of CA_C2195 we provide a view into the possible functions of the protein. From contextual information from gene-neighborhood analysis, we propose that rather than being a peptidase, CA_C2195 and its homologs might play a role in biosynthesis of a modified cell-surface carbohydrate in conjunction with several sugar-modification enzymes. These results provide the groundwork for the experimental verification of the function.

## Background

CA_C2195 from *Clostridium acetobutylicum* [UniProtKB:Q97H19_CLOAB] is a novel 434-residue protein of unknown function. Initial sequence analysis suggested that this protein could be a metallopeptidase. A PSI-BLAST [1] search against UniProt revealed that there are over 200 other similar proteins of unknown function. Pairwise sequence identities of these proteins to CA_C2195 vary between 40-60%. We present here the crystal structure of CA_C2195, determined as part of the Protein Structure Initiative program to extend structural coverage of novel protein sequence space to provide structure-based function assignment [2,3]. CA_C2195 was specifically targeted by the Joint Center for Structural Genomics (JCSG) in an effort to increase the structural coverage of proteins in Pfam [4] clan CL0035 of metallopeptidases (Peptidase MH/MC/MF), which has ~64000 protein sequences (including CA_C2195) in 12 families (Pfam v27.0, March 2013) but with only limited (~0.2%), biased structural coverage. The families that form this clan contain many sequences, are functionally diverse, and are important in numerous biological processes. For example, recombinant bacterial carboxypeptidase G2 is used in cancer therapy to hydrolyze methotrexate [5] and is being tested in prodrug therapy; and human aspartoacylase is implicated in Canavan’s disease in the brain [6]. There are also non-peptidase homologs of these proteins: some of these have active catalytic domains, but perform distinct albeit related enzymatic functions, such as the glutaminyl-peptide cyclotransferase. In other cases the homologous domains are not catalytically active and they perform protein-protein interaction based functions, such as the transferrin receptor proteins 1 and 2. JCSG has determined ~20 structures to date from clan CL0035 (see http://www.topsan.org/Groups/Zinc_Peptidase). Proteins in these families [7,8] have a broad phylogenetic spread across all kingdoms of life and show substantial sequence divergence.

The structure of CA_C2195 revealed that it is composed of three domains. Our sequence and structure analysis led to the assignment of these three domains of CA_C2195 and its homologs to new Pfam families (using standard Pfam protocols) [4], to be released in the next Pfam update, version 28.0: the N-terminal metallopeptidase-like domain to DUF4910 (Domain of Unknown Function, [Pfam:PF16254]), which is distantly related by sequence to the Peptidase_M28 family [Pfam:PF04389] in clan CL0035 (MEROPS [9] M28 family in the peptidase MH clan); the insert domain to DUF2172 [Pfam:PF09940] (a reassignment of the existing entry); and the C-terminal wHTH to HTH_47 [Pfam:PF16221]. We believe that our results may aid in the design of structure-based biochemical experiments to further explore the biology of these proteins similar to other recent efforts on proteins of unknown function [10-15]. Based on a recent study, many DUF proteins are likely essential proteins [16].

## Results and discussion

### Overall structure

**Figure 1:**
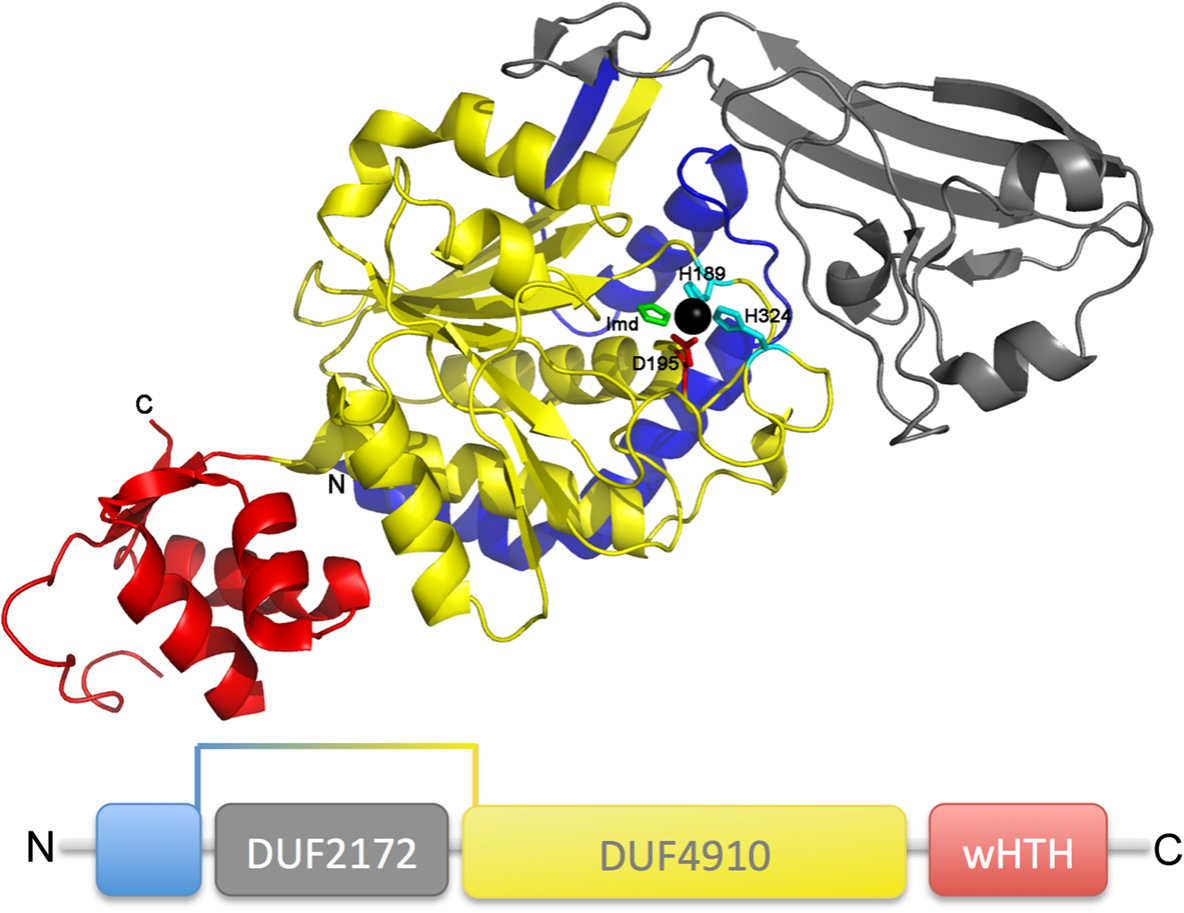
**Crystal structure and domain architecture.** The crystal structure of CA_C2195 from *Clostridium acetobutyliticum*, with the N- and C-termini labeled as ‘;N’ and ‘;C’, reveals 3 domains: residues 1–55 (blue) and 165–355 (yellow) form the N-terminal metallopeptidase domain, DUF4910; residues 56–164 (grey) form the DUF2172 domain; and residues 356–434 (red) form a C-terminal wHTH domain, HTH_47. Residues in the putative active site are Asp195 (red stick); and His189 and His324 (cyan sticks), and they are bound to a Zn ion from the crystallization condition. Imidazole from the crystallization condition is also bound to the active site Zn. The lower panel is a linear representation of the domain architecture of CA_C2195.

The protein production and crystallization of CA_C2195 was performed by standard protocols in the JCSG High-Throughput Structural Biology pipeline (http://www.jcsg.org) as briefly described in Methods. The crystal structure was determined to 2.37 Å by Multi-wavelength Anomalous Diffraction (MAD) phasing and atomic coordinates and experimental structure factors have been deposited in the Protein Data Bank (http://www.wwpdb.org) with PDB accession code 3k9t. Data collection, model and refinement statistics are summarized in Table 1[17-20]. There is one molecule of CA_C2195 in the crystallographic asymmetric unit (Figure 1), which contains 422 of the 434 residues in the entire protein as well as Gly0 that remains after cleavage of the protein expression and purification tag. Residues 374–386 were disordered in the structure and were excluded from the protein model. A zinc ion (Zn) was modeled at the putative peptidase active site based on presence in the crystallization condition as well as an anomalous difference Fourier map. An imidazole molecule (Imd) from the crystallization condition was also modeled based on electron density to coordinate with the Zn. Other solvent molecules include two chloride ions and four (4R)-2-methylpentane-2,4-diol (MRD) molecules from the crystallization condition as well as water molecules. Sequencing of the cloned construct indicated that residue Pro309 was substituted with a serine residue, which was supported by electron density. Based on crystal packing analysis, using the ‘;Protein interfaces, surfaces and assemblies’ service PISA (http://www.ebi.ac.uk/pdbe/prot_int/pistart.html) [21] at the European Bioinformatics Institute (EBI), the predicted biological assembly of CA_C2195 is a trimer. Size-exclusion chromatography coupled with static light scattering, performed during protein production and crystallization screening, also supports a protein trimer in solution. A search for other proteins that may share overall structural similarity to CA_C2195, using the Protein structure comparison service Fold at EBI (http://www.ebi.ac.uk/msd-srv/ssm) [22] produced no significant hits. Examination of the structure revealed three distinct domains: a Peptidase_M28-like metallopeptidase domain with a small α + β domain inserted into it and a C-terminal wHTH domain [23,24].

**Table 1:** Summary of crystal parameters, data collection and refinement statistics for PDB 3k9t

	**λ**_**1 **_**MAD-Se**	**λ**_**2 **_**MAD-Se**	**λ**_**3 **_**MAD-Se**
Data collection			
Space group	H32		
Unit cell parameters (Å)	a = 153.78, b = 153.78, c = 168.38		
Wavelength (Å)	0.91837	0.97925	0.97911
Resolution range (Å)	29.1-2.37	29.1-2.44	29.1-2.25
	(2.43-2.37)	(2.50-2.44)	(2.31-2.25)
No. of observations	172,585	157,212	403,378
No. of unique reflections	31,178	28,543	36,347
Completeness (%)	99.9 (100.0)	99.9 (100.0)	100.0 (100.0)
Mean *I/σ (I)*	9.0 (1.5)	9.2 (1.6)	12.7 (1.9)
*R*_*merge*_ on *I*^†^ (%)	18.9 (101.7)	18.3 (93.1)	20.9 (132.1)
*R*_*meas*_ on *I*^‡^ (%)	20.9 (112.3)	20.3 (102.9)	21.9 (138.4)
*R*_*p.i.m.*_ on *I*^‡‡^ (%)	8.8 (47.4)	8.6 (43.4)	6.5 (41.2)
Model and refinement statistics			
Resolution range (Å)	29.1-2.37		
No. of reflections (total)	31,177^§^		
No. of reflections (test)	1576		
Completeness (%)	100.0		
Data set used in refinement	λ_1_		
Cutoff criteria	|F| > 0		
*R*_*cryst*_^¶^	0.171		
*R*_*free*_^¶^	0.212		
Stereochemical parameters			
Restraints (RMSD observed)			
Bond angles (º)	1.61		
Bond lengths (Å)	0.015		
Average isotropic *B* value^††^ (Å^2^)	29.5		
ESU^‡‡‡^ based on *R*_*free*_ (Å)	0.18		
Protein residues/ atoms	422 / 3386		
Waters / Zn/ Cl/ Imd/ MRD	221 / 1 / 2 / 1 / 4		

Values in parentheses are for the highest resolution shell.

^†^*R*_*merge*_ = Σ_*hkl*_Σ_*i*_|*I*_*i*_*(hkl) - (I(hkl))*|/Σ_*hkl*_ Σ_*i*_*(hkl)*.

^‡^*R*_*meas*_ = Σ_*hkl*_[*N/(N*-1)]^1/2^Σ_*i*_|*I*_*i*_*(hkl) - (I(hkl))|/*Σ_*hkl*_Σ_*i*_*I*_*i*_*(hkl)*[17].

^‡‡^*R*_*p.i.m*_ (precision-indicating *R*_*merge*_) = Σ_*hkl*_[(1/(*N*-1)] ^½^ Σ_*i*_|I_*i*_ (*hkl*) - < I(*hkl*) > | / Σ_*hkl*_Σ_*i*_ I_*i*_(*hkl*) [19,20].

^§^ Typically, the number of unique reflections used in refinement is slightly less than the total number that were integrated and scaled. Reflections are excluded owing to systematic absences, negative intensities and rounding errors in the resolution limits and unit-cell parameters.

^¶^*R*_*cryst*_ = Σ_*hkl*_||*F*_obs_| - |*F*_calc_||/Σ_*hkl*_|*F*_obs_|, where *F*_calc_ and *F*_obs_ are the calculated and observed structure-factor amplitudes, respectively. *R*_*free*_ is the same as *R*_*cryst*_ but for 5.1% of the total reflections chosen at random and omitted from refinement.

^††^ This value represents the total *B* that includes TLS and residual *B* components.

^‡‡‡^ Estimated overall coordinate error [18].

### N-terminal metallopeptidase-like domain (DUF4910)

**Figure 2:**
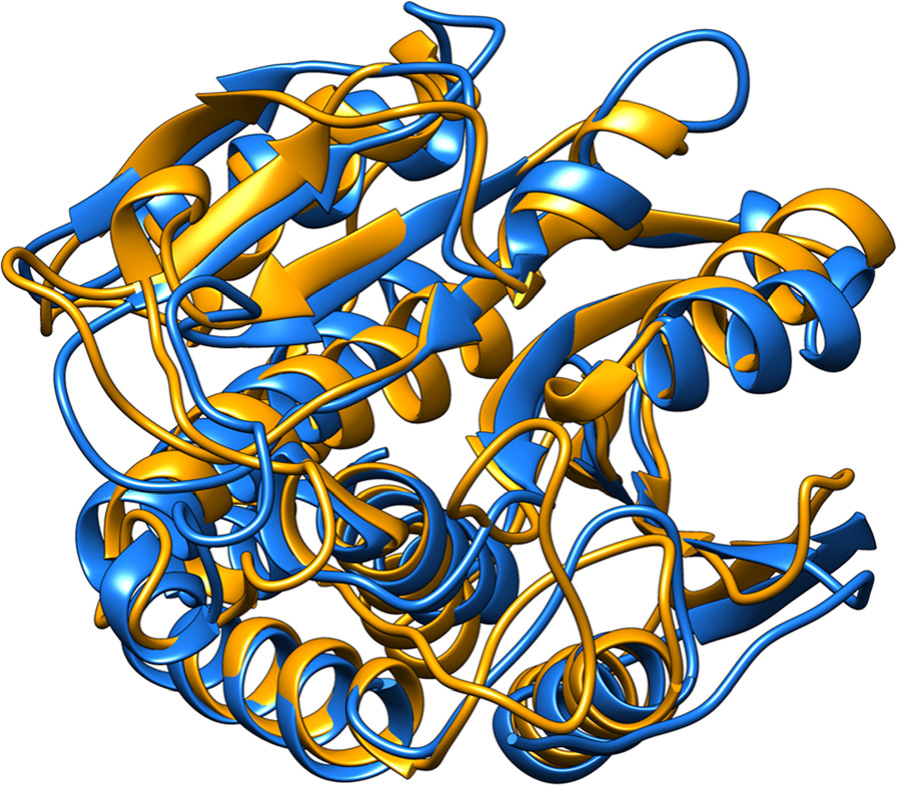
**Metallopeptidase domain structure.** The metallopeptidase domain of CA_C2195 (blue) is similar in structure to several other metallopeptidases, as for example, the Peptidase_M28 family aminopeptidase [PDB:2dea] (orange) with r.m.s.d. ~2.3 Å between C_α_ atoms over the entire domain despite a very low sequence identity of ~17%.

**Figure 3:**
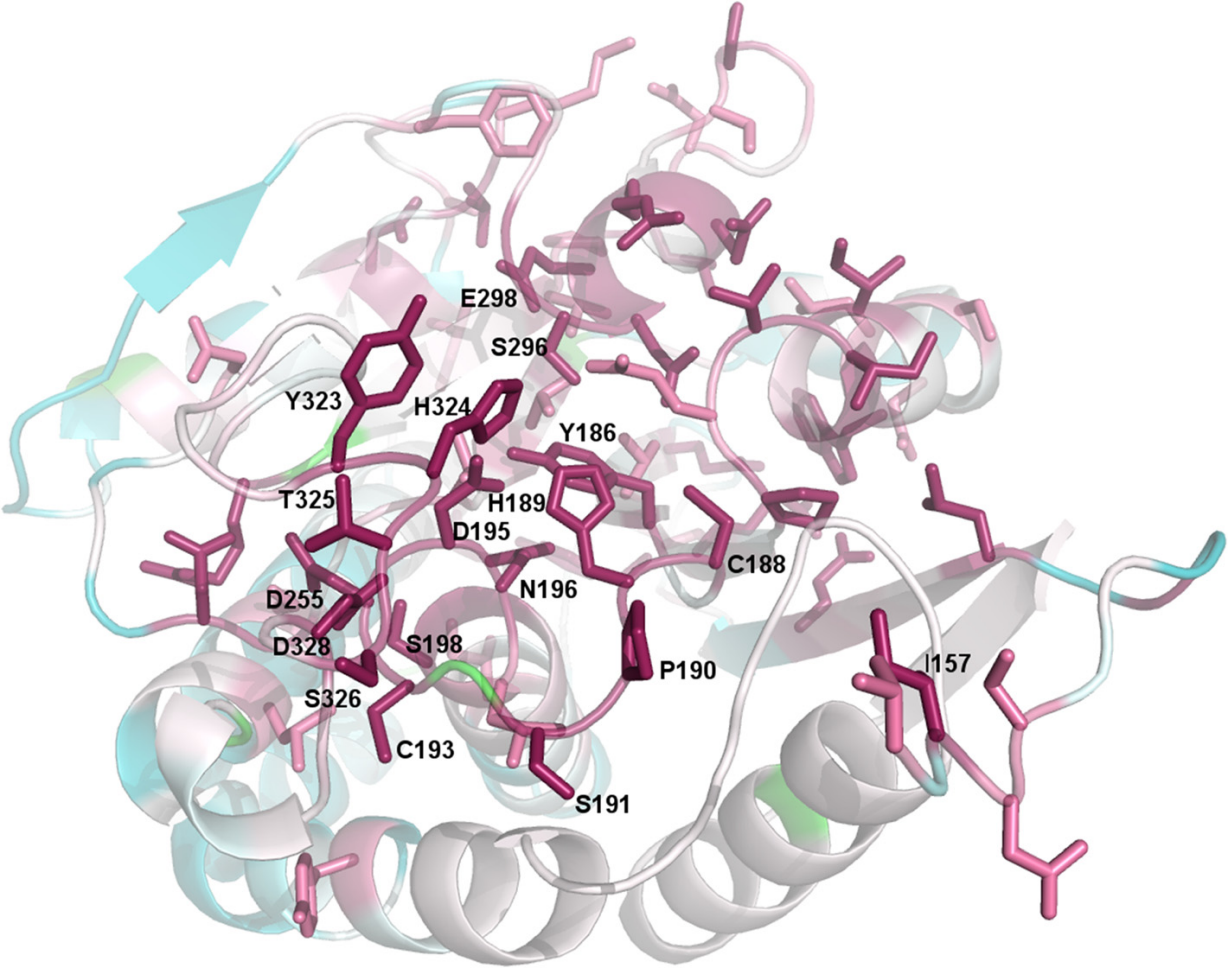
**Residue conservation analysis in the metallopeptidase domain.** The residues likely involved in activity are Asp195, His189 and His324 and have the highest conservation (dark pink, scale 9 in a range of 1 to 9 in CONSURF) across CA_C2195 homologs. The presence of other highly conserved residues around the putative active site suggests that they will also be involved in function. The least conserved residues (cyan, scale 1) in CA_C2195 are also visible.

Out of the 434 residues in CA_C2195, approximately residues 1–55 and 165–355 form the metallopeptidase-like domain, forming the portion that is related to the Peptidase_M28 family [Pfam:PF04389]. A search for other structurally related proteins using Fold produces significant hits to several aminopeptidases (SSM Q-score ~0.4, root-mean-square deviation (r.m.s.d.) ~2.3 Å between C_α_ atoms over the entire domain) with PDB codes [PDB:2dea] (Figure 2), [PDB:1rtq], [PDB:2iq6] and [PDB:3b3t], all structures from the Peptidase_M28 family. However, despite the degree of structural conservation, the level of sequence identity is very low (~17%). The putative active site includes a Zn coordinated with residues Asp195, His189, His324 and the N3 atom from the Imd. It is possible that Imd mimics a portion of the physiological ligand. To identify conserved residues and any potential clustering of such residues, we aligned 82 homologs (ranging from 35-60% sequence identity) and used the conservation profile to mark-up the structure corresponding to DUF4910 (Figure 3). This sequence conservation analysis identified a cluster of conserved residues located within a cleft of the structure, which include Asp195, His189 and His324 that coordinate to the Zn, and together form a putative active site.

All known Peptidase_M28 members bind two Zn ions, which are described as “co-catalytic” as both Zn ions participate in the catalytic activity. In contrast, CA_C2195 has one bound Zn ion. In an earlier study, it was found that HmrA [PDB:3ram] [25], a Peptidase_M20 [Pfam:PF01546] protein (M20 and M28 peptidases are both in the MH clan and closely related to each other), also contained only one Zn ion and that this might have been enough to change its specificity from that of an exopeptidase (aminopeptidase or carboxypeptidase, which are the predominant specificities in both M20 and M28) to that of an endopeptidase. Despite only one Zn ion in HmrA (it is not fully clear whether the HmrA physiologically contains only one Zn ion or whether this was an artifact of the crystallization and that two Zn should be present), all five Zn-coordinating residues expected in Peptidase_M20 are conserved, which is not the case with CA_C2195. In CA_C2195 only the residues that bind the single Zn ion have been retained.

CA_C2195 does not possess conventional Peptidase_M28 active site residues, as both of the essential, invariant, active site residues have been replaced: Ser191 replaces the conserved Asp and Pro225 replaces the conserved Glu. Ser191 is conserved as Ser in 73 of the 82 homologs that were aligned and present as either Ala or Gly in the remaining 9 homologs. Pro225 is conserved as Pro in 81 of the homologs and present as Val in 1 homolog. All enzymes in Peptidase_M28, the closest known peptidase family by structure and sequence, have these residues conserved. There are over 550 non-peptidase M28 homologs in MEROPS, but only a few have been characterized. Those that have been characterized have evolved different functions, for example, the transferrin receptor proteins 1 and 2, and glutaminyl-peptide cyclotransferase. The glutaminyl-peptide cyclotransferase also has all five Zn-binding and both active site Asp and Glu residues conserved [26], therefore, CA_C2195 is unlikely to have comparable catalytic activity. Transferrin in blood serum binds iron, which is internalized once transferrin docks to its receptor [27].

### Insert domain (DUF2172)

**Figure 4:**
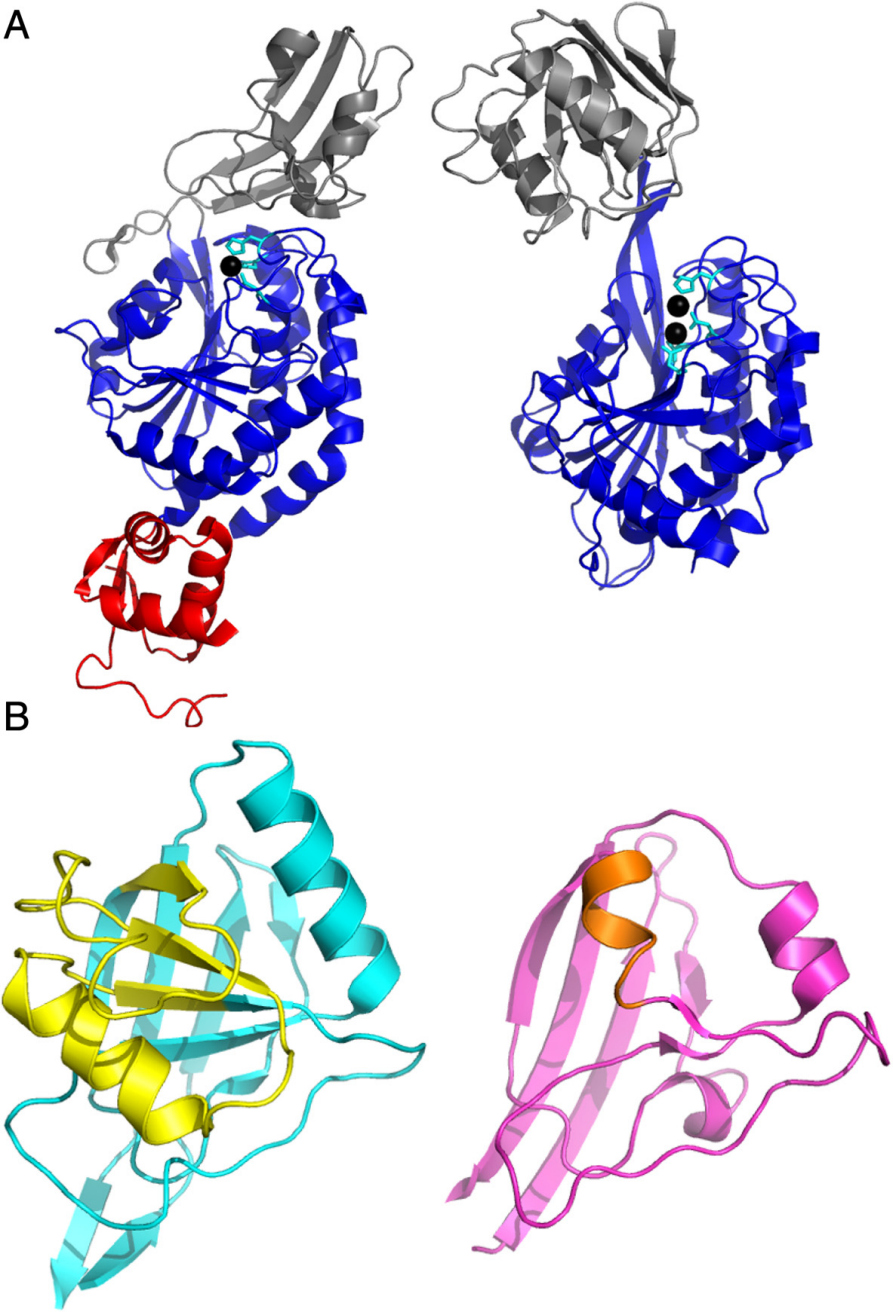
**Comparison of the DUF2172 and PA domains.(A)** The DUF2172 domain in CA_C2195 (grey, left panel) bears some fold resemblance to the PA (Protease-associated) domain (grey, right panel), which has been observed in a Peptidase_M28 family member [PDB:2ek8, right panel) even though there is no discernible sequence identity. Analogous to the proposed role of the PA domain, the DUF2172 domain may be forming a lid modulating access to the peptidase active site and may also be involved in substrate recognition and specificity. Molecules in the panels are oriented such that the peptidase domains in both superimpose. The active sites in both molecules are shown in cyan sticks and black spheres. **(B)** A large substructure of the PA domain fold (yellow, left panel) is replaced with a turn of α-helix in DUF2172 (orange, right panel).

Residues 56–164 (approximately) in CA_C2195 form a separate globular domain inserted into the DUF4910 domain. This insert domain adopts an α+β fold that does not closely match any other known structures. However, careful visual inspection shows (Figure 4) that the insert domain bears a resemblance to the “Protease-associated” domain (PA domain, [Pfam:PF02225]) in terms of gross structure and orientation of insertion. A comparison of the CA_C2195 structure with the structure of an aminopeptidase from *Aneurinibacillus sp*. strain AM-1 [PDB: 2ek8], suggests that its DUF2172 domain is very likely derived from the PA protein domain family (Figure 4). The PA domain is similarly found inserted within several other peptidase domains, which are catalytically unrelated to each other. Interestingly, the PA domain is found inserted in some Peptidase_M28 domains at a structurally equivalent site to that of DUF2172 in DUF4910. It has been suggested that the PA domain may act as a lid, which covers the active site and may be involved in protein recognition in vacuolar sorting receptors [28]. The PA domain of aminopeptidase has a characteristic “swivelling” β/β/α domain fold [24]. In the DUF2172 domain in CA_C2195, there is a turn of an α-helix instead of a large β‒α‒β‒α‒β substructure on one side of the PA domain fold, whereas the remaining structures of the two domains retain overall similarity and differ only by a few minor insertion or deletions (Figure 4). Given their equivalent location relative to the peptidase domain, we propose that the DUF2172 domain has probably evolved from the PA domain in a pre-existing multi-domain context, that is, after its merger with the catalytic domain.

**Figure 5:**
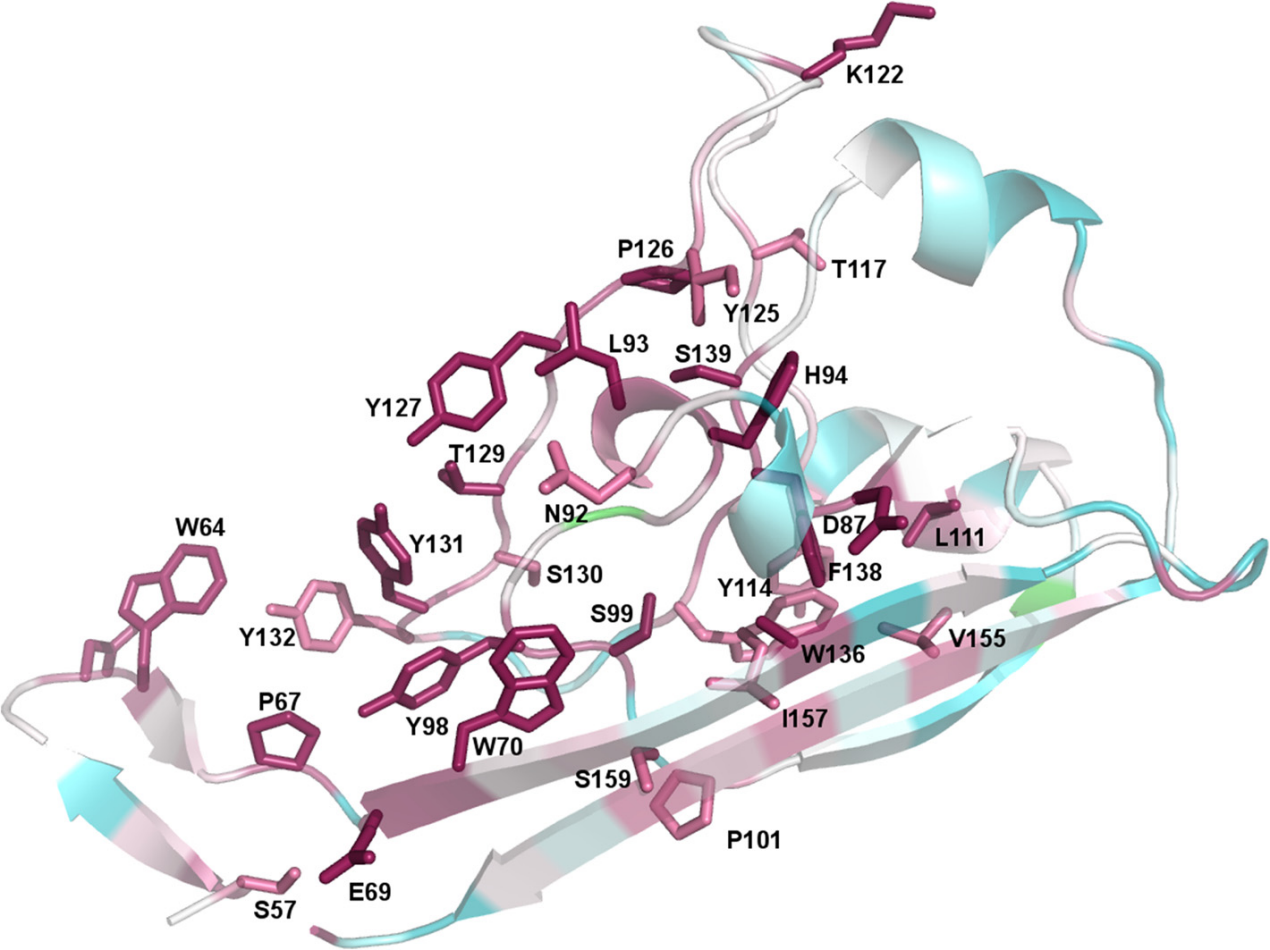
**Residue conservation analysis in the DUF2172 domain.** The presence of highly conserved aromatic residues (dark pink) including Trp70, Tyr98, Tyr127, Tyr131 and Tyr132, indicates residues that may be involved in substrate recognition if this domain has a functionality associated with substrate interactions.

To study sequence conservation in DUF2172 homologs, thereby allowing the identification of residues that may be functionally important, 80 sequences ranging in identity from 47-66% were aligned and the conservation profile used to mark-up the structure corresponding to DUF2172 (Figure 5). Numerous aromatic amino acid residues appear to be the most conserved in this domain: Trp70, Tyr98, Tyr127, Tyr131 and Tyr132. Speculatively, these residues might be important in binding to target proteins if, like the PA domain, this domain is involved in protein recognition.

### C-terminal wHTH domain (HTH_47)

**Figure 6:**
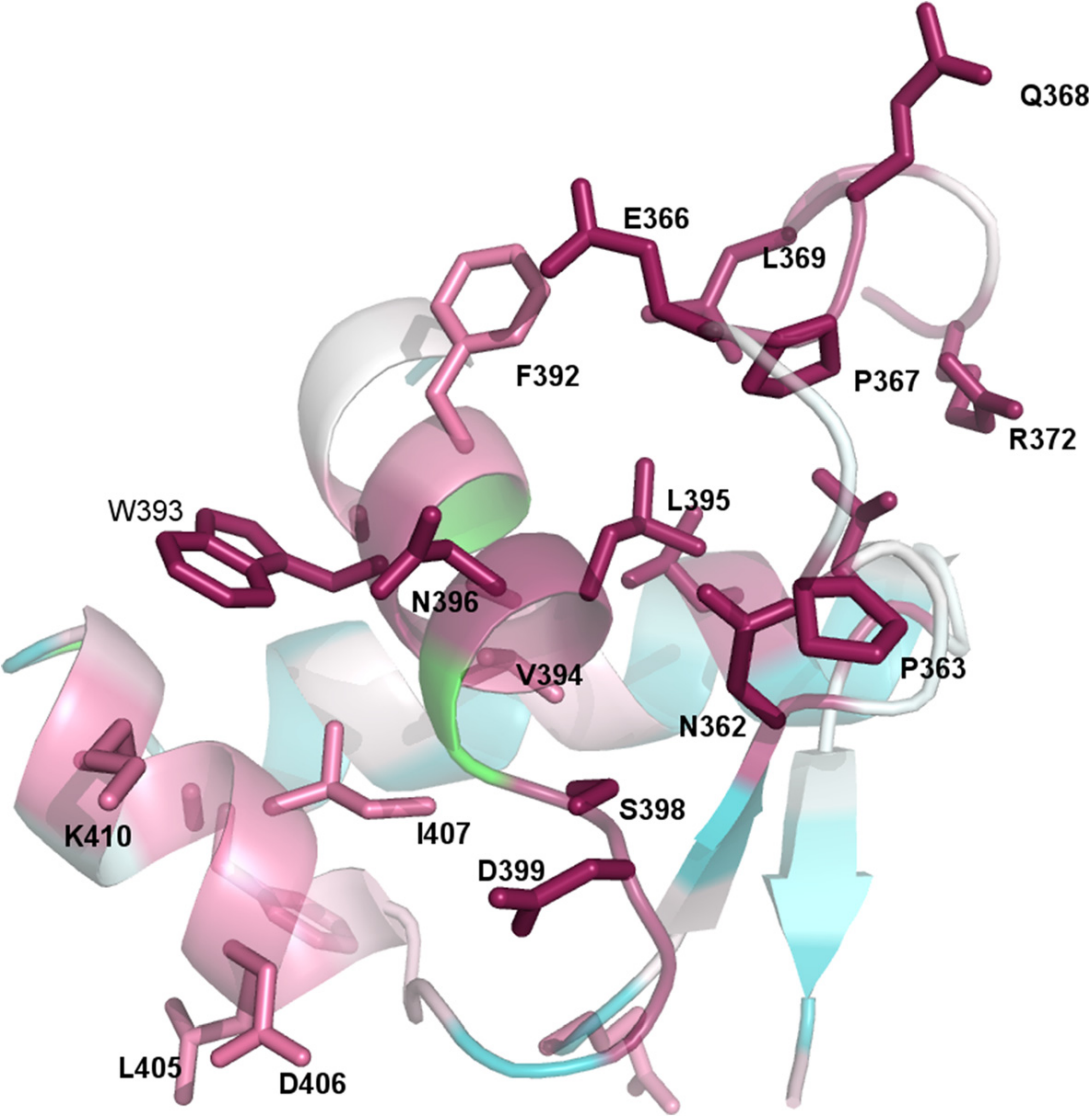
**Residue conservation analysis in C-terminal wHTH domain.** Residues in the C-terminal circularly permuted wHTH domain that might be involved in substrate recognition and specificity based on their high conservation across CA_C2195 homologs (residues with highest conservation are in dark pink) are visualized.

One of the most interesting aspects of CA_C2195 and its homologs is the presence of a unique C-terminal circularly permuted wHTH domain in conjunction with the metallopeptidase domain. A search for other proteins using Fold that are similar to this domain (residues 356–434) results in very significant hits (SSM Q-score ~0.4, r.m.s.d ~2.0 Å between C_α_ atoms over the entire domain) with other wHTH domains, although the sequence identities of these hits are in the 15-19% range (the PDB codes of the top 4 hits are: [PDB:2xvc], [PDB:2yu3], [PDB:1cf7], [PDB:3o6b]). A Jackhmmer [29] search using default search parameters identifies matches on the third iteration to sequences corresponding to the position of MarR_2 [Pfam:PF12802] transcription factors. Structures of sequences belonging to MarR_2 also adopt a wHTH topology, supporting the structure-based search at the sequence level, but clearly show that this wHTH has diverged in terms of sequence from other known wHTH domains. To identify residues that may be functionally important based on sequence conservation, 43 homologs ranging in sequence identity from 36%-79% were used, out of which only one sequence had higher than 53% sequence identity (Figure 6). This revealed that residues with the highest conservation are surface exposed in this domain, suggesting that their role may be in surface-mediated contacts.

**Figure 7:**
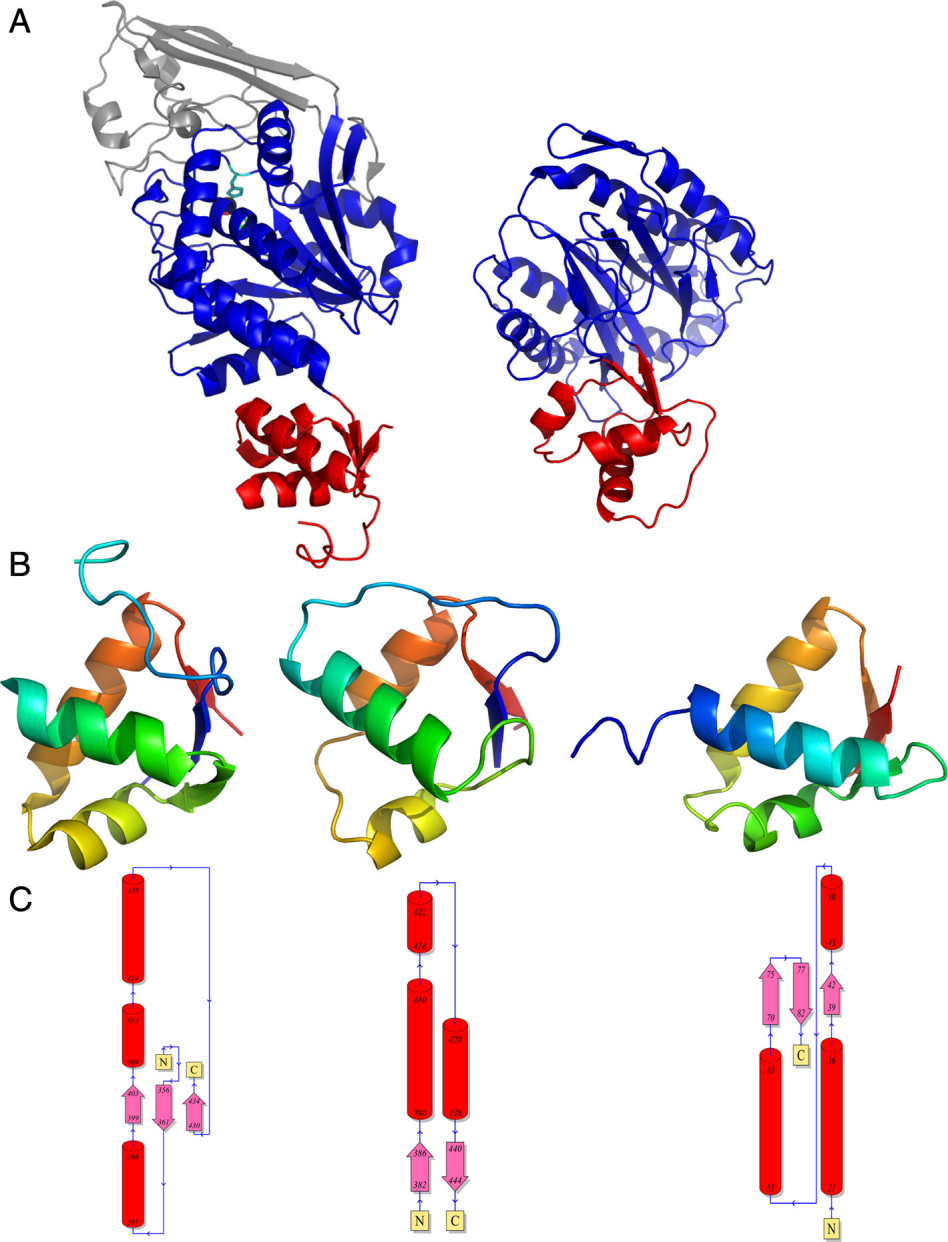
**Comparison of wHTH domains.(A)** The circularly permuted wHTH domain observed in CA_C2195 (red, left panel) resembles another circularly permuted wHTH domain present in the structure of a Peptidase_M24 family aminopeptidase [PDB:1boa] (red, right panel), and may be involved in substrate recognition and specificity. **(B)** The wHTH domain in CA_C2195 (left) is compared to the wHTH domain from Peptidase_M24 [PDB:1boa] (center) and a wHTH domain from a transcription factor [PDB:1cf7] (right), which was one of the proteins most similar in structure to the CA_C2195 wHTH domain. Each domain is colored from the N-terminus (blue) to the C-terminus (red). All domains are in a similar orientation. **(C)** Topology diagrams for the three domains in (B) in the same order depicting the arrangement of secondary structure elements and circular permutation in the CA_C2195 wHTH compared to the transcription factor wHTH. Cylinders represent α-helices, arrows represent β-strands and the N- and C-termini are labeled.

The juxtaposition of a metallopeptidase with a wHTH domain is not common, although a similar domain architecture has been observed previously in methionine aminopeptidase-2 (Met-AP2). The wHTH domain in Met-AP2 is inserted within a distinct peptidase domain belonging to the Peptidase_M24 family [Pfam:PF00557], which includes the creatinases and prolidases. In Met-AP2, the inserted wHTH domain has been shown to be important for the recognition and specificity of the substrate, namely, the amino-termini of proteins processed by the enzyme [30] [PDB:1boa]. Interestingly, comparison of the CA_C2195 and Met-AP2 wHTH domains indicates that they have a similar permutation of the wHTH domain (Figure 7). Furthermore, as in the case in the Met-AP2, the CA_C2195 wHTH domain is spatially located as a distinct module, which points away from the core catalytic domain. Thus, by analogy to the Met-AP2, we propose that the permuted wHTH might serve in a similar capacity in substrate recognition and specificity in CA_C2195 and its homologs. In a more general sense, the recognition of circularly permuted domains independently fused to two distinct classes of peptidases raises the possibility that these domains may have been more generally recruited as potential peptide-recognition modules early in the history of proteins.

### Oligomeric assembly

As mentioned above, crystal packing analysis predicts a trimer as the oligomeric form in solution, which is supported by size-exclusion chromatography coupled with static light scattering. The trimeric assembly is formed by the interaction of residues in the wHTH domain (loop residues 362–368 and helix residues 389–393) with loop residues 62–64 in the insert domain and loop residues 302–305 and 293–294 in the metallopeptidase-like domain. Some of these residues forming the assembly in all 3 domains show high conservation, indicating that these are likely to be the key binding residues in the protein interaction interface. In particular, a substantial portion of the surface on one side of the wHTH appears to be responsible for mediating the monomer protein interactions in the oligomeric state, covering the majority of the highly conserved residues. These observations strongly suggest that the wHTH functions in mediating protein interactions in the oligomeric state.

### Conserved gene neighborhoods point to a potential role in modified carbohydrate biosynthesis

As described above, the sequence and structural analysis indicates that the conserved residue pattern does not conform to any known peptidase active site. Therefore, to better understand the possible biochemical function of CA_C2195, we used contextual information gleaned from conserved gene neighborhoods. Several studies have shown that genome context or conserved gene-neighborhoods provide information in terms of functionally interacting partners or complexes to which particular proteins belong [31-33]. Interestingly, we found a strong gene-neighborhood association (and in some cases gene fusions) between CA_C2195 and its homologs with several genes involved in biosynthesis of a modified carbohydrate across several phylogenetically distinct bacterial taxa, namely actinobacteria, firmicutes, cyanobacteria, bacteroidetes, planctomycetes (Table 2, Additional file 1, Additional file 2). This wide phyletic spread of the association suggests that the co-occurrence is likely to be of functional importance for these enzymes. Among the strongly linked genes we found those coding for a sugar epimerase/dehydratase, a sugar phosphate nucleotidyltransferase, a glycosyl transferase, an aminosugar *N*-acetyltransferase and a SAM-dependent sugar methylase. These enzymes are all associated with carbohydrate metabolism, and are indicative that a modified sugar is being synthesized by the action of multiple enzymes and converted to a nucleotide diphosphate linked sugar by the action of the nucleotidyltransferase. This NDP-sugar then probably serves as the substrate for the glycosyltransferase that transfers it to a target moiety. However, examination of the predicted operons also reveals variability especially in terms of the numbers of genes encoding for glycosyltransferases, sugar methylases and other auxiliary modifying enzymes such as those that act on sugars to add acyl groups (Table 2, Additional file 1, Additional file 2).

**Table 2:** Gene neighborhood analysis

**GI number**	**Gene**	**Locus**	**Protein**	**Product**
15895455	.	CA_C2186	NP_348804.1	Glycosyltransferase
15895456	spsE	CA_C2187	NP_348805.1	N-acetylneuraminic acid synthase + SAF sugar-binding (condenses of phosphoenolpyruvate and *N*-acetylmannosamine)
15895457	.	CA_C2188	NP_348806.1	Glycosyltransferase
15895458	.	CA_C2189	NP_348807.1	ATP-grasp amino acyl ligase
15895459	spsF	CA_C2190	NP_348808.1	Sugar phosphate nucleotidyltransferase
15895460	.	CA_C2192	NP_348809.1	Glyoxylase
15895461	.	CA_C2193	NP_348810.1	DUF3880 + Glycosyltransferase
15895462	.	CA_C2194	NP_348811.1	nucleoside-diphosphate sugar epimerase
*15895463*	*.*	*CA_C2195*	*NP_348812.1*	*Peptidase-like (peptidase_MH superfamily)*
15895464	.	CA_C2196	NP_348813.1	Methyltransferase + Glycosyltransferase (currently annotated as: MAF_flag10, DUF115)
15895465	.	CA_C2197	NP_348814.1	aminosugar *N*-acetyltransferase
15895466	acpA	CA_C2198	NP_348815.1	acyl carrier protein
15895467	.	CA_C2199	NP_348816.1	aminosugsar *N*-acetyltransferase + HAD Phosphatase

This linkage between a gene coding for a peptidase-like protein with a carbohydrate biosynthetic system could be explained in at least three alternative ways: 1) CA_C2195 protein and its homologs are post-translationally glycosylated; 2) The DUF4910 domain cleaves target proteins alongside their modification by glycosylation; 3) The DUF4910 domain actually participates in the biosynthesis of a sugar-derived metabolite by catalyzing a reaction biochemically distinct from the classical peptidase reaction. Circumstantial evidence supports the third alternative. First, as discussed above, the CA_C2195-like genes do not seem to preserve the conventional metallopeptidase active site. Moreover, these genes are usually embedded in the middle of an operon with genes for carbohydrate-modifying enzymes on either side. Second, these operons do not show any linked genes coding for other potential target proteins. Third, in several cases these operons contain genes for a transmembrane carbohydrate export protein (related to the O-antigen and teichoic acid export proteins) and transmembrane sugar pyruvyltransferase (Table 2, Additional file 1, Additional file 2). These proteins suggest that the modified carbohydrate is unlikely to be used to modify intracellular proteins; rather it is likely to be translocated to the cell-surface and used as part of a surface polysaccharide/lipopolysaccharide. In light of these observations it is possible that DUF4910 is involved in modification of the sugar-derived metabolites, perhaps via transacylation of a peptide/glutamine to an amino sugar. In principle, they could also be used in an amidase reaction for deacylation of a sugar amide, but this would imply that they utilize distinctive active site residues (see above). TMPRED (http://www.ch.embnet.org/software/TMPRED_form.html) predicts one significant transmembrane helix in CA_C2195 (residues 192–213, inside to outside, score 557), which is buried in the metallopeptidase-like domain (and therefore incorrectly predicted to be transmembrane), and Phobius [34] predicts most of the protein to be extracellular, with a dip where the possible transmembrane helix might be. SignalP [35] fails to predict a signal peptide and so it is unknown how this protein gets into the periplasm or if it is extracellular.

## Conclusions

The crystal structure of CA_C2195 and subsequent sequence-structure-function analysis shows that CA_C2195 (and ~200 homologs, ranging in sequence identity from 40-60%) is a three-domain protein, which includes a C-terminal wHTH domain and a DUF2172 domain inserted in the DUF4910 metallopeptidase-like domain. The presence of the PA domain-like DUF2172 domain shows similarity in domain architecture to some members of the Peptidase_M28 family [PDB: 2ek8]. However, the presences of a C-terminal wHTH domain in CA_C2195, shows similarity to domain architectures found in Peptidase_M24 [PDB:1boa]. Analysis of sequence conservation reveals a cluster of non-sequential, highly conserved residues on the surface of the structure of CA_C2195, which are likely to be functionally important, some of which in the wHTH are involved in forming the protein interaction interface in the oligomeric form. It is possible that these proteins do not have any metallopeptidase activity because of the absence of all the catalytic residues that are expected from other characterized members of this peptidase clan. Based on gene neighborhood analysis, we propose that CA_C2195 and its homologs could be involved in the biosynthesis of modified carbohydrates. Given the importance of cell surface polysaccharides in inter-organismal interactions, further characterization of the biochemical activity of this protein is likely to be of interest in the case of pathogens that encode a CA_C2195 like gene, such as *Brucella* and *Campylobacter*.

## Methods

Protein production and crystallization of CA_C2195 was carried out by standard JCSG protocols [36-38]. Data collection was performed at SSRL beamline 9–2. The crystal structure was determined by MAD phasing using a seleno-methionine-derivatized protein. X-ray data collection, processing, structure solution, tracing, crystallographic refinement and model building were performed using BLU-ICE [39], MOSFLM [40]/SCALA [41], SHELXD [42]/AUTOSHARP [43], ARP/wARP [44], REFMAC [45] and COOT [46]. To find homologs for sequence conservation analysis, PSI-BLAST was used to search the Uniref90 database in 3 iterations with e-value cutoff of 0.0001, searching for a maximum of 150 homologs between 35-95%, using MAFFT as the alignment method MAFFT, Bayesian calculation method, and JTT evolutionary substitution method, as implemented in CONSURF [47]. Figure 2 was prepared using Chimera (http://www.cgl.ucsf.edu/chimera) and all others were prepared using PyMOL [48]. The topology diagrams in Figure 7C are from PDBsum [49]. Gene neighborhood was comprehensively analyzed using a custom Perl script using the *CA_C2195* gene or its homolog as anchors. This script uses either the PTT file (downloadable from the NCBI ftp site) or the Genbank file in the case of whole genome shot gun sequences to extract 20 gene neighbors on the 3’ and 5’ sides of a given query gene. The protein sequences of all neighbors were clustered using the BLASTCLUST program (ftp://ftp.ncbi.nih.gov/blast/documents/blastclust.html) to identify related sequences in gene neighborhoods. Each cluster of homologous proteins were then assigned an annotation based on the domain architecture or conserved shared domain which were detected using Pfam models and in-house profiles run using RPS-BLAST [50]. This allowed an initial annotation of gene neighborhoods and their grouping based on conservation of neighborhood associations. In further analysis, care was taken to ensure that genes are unidirectional on the same strand of DNA and shared a putative common promoter to be counted as a single operon. If they were head to head on opposite strands they were examined for potential bidirection promoter sharing patterns. A total of 4789 representative bacterial and archaeal genomes were analyzed for the detection of CA_C2195 orthologs. These genomes spanned representatives of all currently known major lineages of bacteria and archaea. From these 229 genomes were identified as having CA_C2195 orthologs with gene neighborhoods and further analysis was performed on this subset of genomes. Within this subset conserved gene neighborhood associations were detected in 10 major bacterial clades namely actinobacteria, firmicutes, cyanobacteria, planctomycetes, bacteroidetes, nitrospirae, alphaproteobacteria, betaproteobacteria, epsilonproteobacteria and spirochaetes. Using a simulation with sampling with no replacement and the average genome size of 4000 genes we found that such genes as described above coming together by chance alone in such neighborhoods was p < 10^-9^. For all bioinformatics analyses that were performed using homologs within a family for comparison, the chosen sequences were well over the inclusion threshold for the family as built.

### Availability of supporting data

Atomic coordinates and experimental structure factors for CA_C2195 have been deposited in the Protein Data Bank (http://www.wwpdb.org) with PDB accession code 3k9t (DOI:10.2210/pdb3k9t/pdb).

## Competing interests

The authors declare that they have no competing interests.

## Authors’ contributions

DD performed x-ray structure determination; DD, LA, AGM, NDR, RDF and PC analyzed the sequence-structure-function relationships and prepared the manuscript; and AB and AG provided assistance on the analysis of DUF proteins and critically reviewed the manuscript. All authors read and approved the final manuscript.

## Supplementary Material

Additional file 1 **Conserved gene neighborhoods with representatives of other bacterial lineages.** Description of data: Table describing genomic context in support of Table 2. Click here for file

Additional file 2 **Gene neighborhoods from all organisms containing an ortholog of CA_C2195.** Description of data: Table describing genomic context in support of Table 2. Click here for file
